# Heart rate variability biofeedback intero-nociceptive emotion exposure therapy for adverse childhood experiences

**DOI:** 10.12688/f1000research.20776.2

**Published:** 2022-04-19

**Authors:** Stéphanie Hahusseau, Bruno Baracat, Thierry Lebey, Lionel Laudebat, Zarel Valdez, Arnaud Delorme

**Affiliations:** 1 Cabinet Médical de Stéphanie Hahusseau, Paris, France; 2 EA SCoTE, IU JF Champollion, Albi, France; 3 LAPLACE, Federal University of Toulouse, Toulouse, France; 4 CERCO, Universite Paul Sabatier, Toulouse, France; 5 Centre de rechercher Cerveau et Cognition, CNRS, Toulouse, France; 6 Institute of Neural Computation, University of California San Diego, Santa Diego, CA, USA; 7 Institute of Noetic Sciences, Petaluma, CA, USA

**Keywords:** Interoception, Adverse Childhood Experience, developmental trauma disorder, Biofeedback, Retrospective study, autonomic nervous system, PTSD

## Abstract

**Background: **Psychiatric patients with adverse childhood experiences (ACE) tend to have dysfunctions in the interoceptive part of their emotional experience. The integration of interoceptive emotional activity in the insular and cingulate cortices is linked to the regulation of sympathovagal balance. This makes heart rate variability (HRV) an ideal measure for providing feedback on emotion regulation in real-time.

**Methods: **A sample of one hundred (n=100) outpatients was evaluated. Participants underwent eight 30-minutes ACE exposure sessions during which patients were guided to experience bodily sensations related to ACE while their HRV was monitored using a commercial biofeedback device.

**Results: **Comparing the results of the first to last therapeutic session, a significant decrease in heart rate and an increase in HRV at the onset of the session were observed.

**Conclusions: **This study suggests a physiological impact of therapeutic interventions on autonomic balance and underlines the interest in HRV biofeedback as clinical practice.

## Introduction

### Heart rate variability

Heart rate variability (HRV) is a measure of variation in time between heartbeats. Heartbeats may occur at regular intervals (low HRV) or irregular intervals (high HRV). Among many other things, low Heart Rate Variability reflects an individual's ability to adaptively cope with stress. According to Thayer’s model ^1^ (see also 2, 3), orthosympathetic activity is associated with higher central nervous system activity, in particular activity within the limbic system, the amygdala, and the prefrontal and frontal cortex ^4^. One of the roles of these high-level structures is to inhibit the parasympathetic system and activate the sympathetic system. When a person faces a threat, this may elicit a hyperarousal and “ *flight or fight”* response ^5^, which leads to an inhibition of the parasympathetic system and an activation of the sympathetic system. This corresponds to a decrease of HRV and often an increase in Heart Rhythm (HR) ^6^. Emotional events may have an influence on the general stress level ^7^, which in turn is visible in the sympathetic/parasympathetic balance. Generally, these effects are transient because the higher nervous structures (essentially amygdala and prefrontal cortex) inhibit each other and, as soon as the stressor disappears, the system returns to parasympathetic tonus with low HR and high HRV.

For several decades, autonomic nervous system tests have been used to identify the physiologic correlates of psychiatric illnesses, particularly for affective and anxiety disorders ^8^. In studies of Post-Traumatic Stress Disorder (PTSD) for example, decreased HRV are observed in PTSD patients compared to matched controls ^9^. The HRV of PTSD patients indicates an increase in sympathetic activity and a reduction in parasympathetic activity. Patients suffering from PTSD tend to exhibit hyperactivity of the autonomous nervous system at rest and have been shown to be unable to further mobilize their orthosympathetic system when facing a stressful situation ^10^. In addition, the HRV profile after exposure to a trauma has been shown to be predictive of future traumatic episodes in PTSD ^11, 12^. PTSD is associated with the disruption of the autonomic processes that maintain heartbeat regulation ^13^.

### Clinical impact and assessment of adverse childhood experiences

Research in psychiatry indicates that adverse childhood experiences leave durable physiological and neurophysiological traces and that there is a strong relationship between adverse childhood experiences and depression, suicide attempts, alcoholism, drug abuse, and other negative health outcomes ^14^. Adverse childhood experiences (ACEs) are ubiquitous among the adult patient population, with about 60% of the population having experienced at least one ^15, 16^. The damaging effects of ACEs are nonspecific, thereby affecting a variety of functions and behaviors. In fact, ACEs have been shown to be negatively correlated with adult mental well-being ^17^. Chronic traumatic experiences in childhood that extend over several years – as in cases with trauma and neglect – impair self-regulation function such as mood regulation and constancy in relations, described in “complex PTSD” and “developmental trauma disorder”.

### Physiological impact of adverse childhood experiences

Clinically, autonomic nervous system (ANS) function and emotional well-being are closely related ^18^. Research has shown that having experienced early-life adverse events was associated with lasting effects on Heart Rate Variability (HRV) ^19^, revealing complex interactions between traumatic experiences, ANS functioning and psychopathology ^20^.

In addition, psychiatric research has shown that having experienced early-life adverse events was associated with altered interoception ^21^. Interoception, defined as the sense of the internal state of the body, is crucial for well-being ^22^ as it mediates emotion regulation ^23^. In fact, most psychiatric disorders are sustained by a type of interoceptive phobia ^24^, i.e. a fear to feel one’s body sensations. Interoception requires the interplay between perception of body states and cognitive appraisal of these states to inform emotional experience and motivating regulatory behavior ^25^. The insular cortex in humans processes interoceptive activity and integrates and modulates cardiovascular, respiratory and emotional signals in order to create an integrated emotional experience ^26^.

### Evidence-based treatment for adults

Neurophysiological impairments due to ACEs have been shown to be reversible ^27, 28^. Evidence-based psychotherapy for adults with ACE history typically involves a progression through three phases: safety and stabilization; trauma processing; consolidation of therapeutic gains ^29^. The trauma processing phase requires sensitive therapeutic guidance. The other phases are best-practice approaches to all psychotherapeutic treatments, with the focus on the unique impact of ACEs.

Evidence based psychotherapy models for adults with ACEs-related disorders such as emotion-focused trauma therapy and eye-movement-desensitization-reprocessing are useful in the trauma processing phase. The efficacy of these approaches may be related to interoception rather than cognitive focusing ^30^. Efficacy of psychotherapy with trauma patients may depend on the patient being able to face and feel adverse sensory and perceptual stimuli related to trauma-related memories in paced conditions ^31^.

Prolonged exposure therapy and cognitive processing therapy have gathered a significant amount of empirical support for PTSD treatment. However, they are not universally effective with patients continuing to struggle with residual post-adverse childhood-traumatic symptoms. As such, another type of intervention such as biofeedback may be beneficial. When patients with PTSD were assigned to receive HRV biofeedback plus treatment, the results indicated that HRV biofeedback significantly increased the HRV while reducing symptoms of PTSD ^32^. The present study intends to replicate these results using commercially available biofeedback equipment within an ecological therapeutic environment.

## Methods

### Patient’s inclusion

The most recent 100 outpatients of therapist SH having experienced at least one type of adverse childhood experience and having used the biofeedback method described above were retained as study population. Only patients for whom more than 3 consecutive sessions were collected were included. These two conditions were the only inclusion criteria. 100 patients was judged appropriate for an HRV study of this nature based on the literature ^33, 34^. In general, HRV studies require about of 100 patients or subjects to observe links between mental conditions and HRV measures, although some studies have observed significant effects in depressive subjects with group sizes as low as 27 ^35^. Inclusion criteria included an history of adverse childhood experience (therapist assessment). Patients who required psychiatric medical treatment involving medication (therapist assessment) were excluded from the study. Patients were included regardless of DSM V guidelines for trauma since these do not provide a definition for patients having experienced chronic trauma over several years such as neglect. However, sub-categories in the DSM V were considered as described in a later section. The data was collected over one year. Table 1 summarizes the main features of the data sample.

**Table 1.** Data sample statistics.

	Men	Women
Number of participants	20	80
Mean age in years	37.5	37.8
Mean number of sessions	6.5	5.5
Mean duration of therapy (in days)	206	201

The clinical assessment of the therapist allowed the creation of the following categories of patients mapped onto DSM V categories: Substance abuse (SA); Somatoform Disorders (SD); Anxious Disorders (AD); Serious Personality Disorder (SPD); Post Traumatic Stress Syndrome (PTSS). All the patients could be diagnosed with trauma complex or developmental trauma disorder ^33^. The diagnosis was established by the therapist based on an interview with the patient. In addition to these categories, additional independent variables were retained: patient age, patient sex, the number of meetings in phase 2 (see below), and the number of days between the first and the last data recording sessions. Data was collected by the therapist on custom forms that were later transcribed into digital form after the anonymization process.

### ACE therapy with interoceptive component

This procedure was developed by therapist MD SH (first author) for over more than 10 years. The therapeutic protocol comprises two parts. In the first part, which typically comprised eight weekly sessions of half an hour each, the therapist (co-author SH) identified the occurrence of adverse childhood experiences (psychological abuse, physical abuse, contact sexual abuse, or exposure to household dysfunction during childhood, e.g. exposure to substance abuse, mental illness, violent treatment by parent or stepparent, criminal behavior in the household). To do so, the therapist carried out clinical investigations, collected anamnestic and diachronic data, and guided the patient to specific breathing visualization exercises.

In order to characterize adverse childhood memories responsible for interoceptive phobia, the patient was asked to initially focus his attention on their breath, then on nociceptive sensations, and finally on the childhood memories ^36^. The therapist asked the patient to focus their attention on their breathing while describing the images associated with the memories and specific body sensations or pain that might arise in detail. This exercise was carried out with closed eyes. During this exercise, each uncomfortable physical sensation and negative thought was rated in terms of intensity on a 10-point scale. Later, after a meeting devoted to the conceptualization of the selected traumatic memories and their influence on repetitive negative emotions, the therapist helped the patient to establish a coherent narrative within which to frame their difficulties. The practitioner explained the therapeutic hypothesis, which would be instantiated in the second phase of the therapy indicated as described below.

The second phase of the therapy consisted of bi-monthly one-hour therapeutic sessions. In each session, after five minutes of rest, the therapist asked the patient to wear an ear device sensor which is part of a HRV biofeedback device (Emwave2; Heartmath, Inc.). The patient was then asked to focus their attention on their breathing for two minutes. After two minutes, the evocation of images related to the adverse childhood memory chosen for this session started ^37^. To avoid dissociative processes and develop interoception and parasympathetic activation, the patient was asked to focus their attention on the uncomfortable bodily sensations for about 30 minutes ^38^. Feedback on the sympathetic-vagal balance was directly affected by the sound delivered by the biofeedback device. The sound of the biofeedback device is correlated with the low-frequency peak in the HRV spectrum (HeartMath Emwave 2 device and associated software; US patent 6,358,201 and Australian patent 770323). The number of sessions depended on the number of adverse childhood experiences to face – in general about 6 sessions. During these meetings, the therapist saved the series of heartbeat intervals (R to R intervals) using the biofeedback software. In this study, five minutes of data at the beginning and at the end of the first session of phase 2 (session 1) and the last session of phase 2 (designated as “session 2” even though there might be several sessions between “session 1” and “session 2”) have been analyzed. Each of these 5 minutes comprises 2 minutes of breathing plus the evocation of traumatic imagery.

### Compliance with ethical standards

The local ethical committee (Comité de protection des personnes Sud Ouest) approved the study and the use of the data for research purposes. Since the study was performed retrospectively, no patient consent was necessary. However, the French national entity for the protection of public and medical digital records (CNIL) authorized the retrospective use of the clinical data for this research (authorization number 1685185). The therapist associated a random number with each patient which was then used to anonymize the questionnaire data, the scanned notes of the therapist, and the EKG files of each patient. Except for the therapist (co-author SH), all other investigators were blind to the identity of the patients. The blinding procedure consisted in assigning a randomly generated code to patients, in compliance with CNIL requirements (Commission nationale de l'informatique et des libertés). It was performed at the therapist’s office by the therapist herself to ensure that no identifiable document could inadvertently be lost, stolen, or read by anyone else than the therapist. When a paper form contained identifiable information, it was masked by the therapist, a sticker with the anonymized patient ID was temporarily placed on the form and the form was photocopied for later digital transfer. The questionnaire data was not integrated into the current report to focus on the interpretation of heartbeat intervals.

### Data collection and data processing

R to R intervals were collected during therapeutic sessions using the biofeedback Emwave2© device. All patients were at rest and sitting during data collection. This system uses a photoplethysmographic sensor located on the right ear lobe and series of heartbeats are automatically extracted by the biofeedback software. The accuracy of this data was verified in one subject by comparison to a simultaneously recorded real EKG (Biopac MP36 unit and Acqknowledge© using Einthoven Lead II derivation): the heartbeats monitored by the biofeedback system were delayed in comparison to the EKG based on the time it takes for blood pressure to build up at the ear lobe. Except for this delay, heartbeat measurements were accurate within millisecond precision in comparison to those visible on the EKG. Using heartbeat time intervals over 300 seconds, HRV calculations were carried out with the Biomedical Toolkit used on Labview© version 2009. This software performs HRV calculations in the same way as other HRV software packages do – such as the popular Kubios software (Kubios Oy, Finland). R to R intervals were resampled at 8hz, and the power spectrum was calculated over the whole 5-minute record using an FFT decomposition. Power was obtained at each frequency by calculating the square value of the FFT absolute amplitude. In the frequency domain, total HRV was obtained by summing the total spectral power for the low-frequency band (LF) 0.05Hz–0.15Hz and the high-frequency band (HF) 0.15Hz–0.35Hz. The LF/HF ratio was also calculated. Before performing statistical analyses, a log (Ln) transformation was applied and values were subsequently normalized across subjects. Other heart measures calculated in the time domain were Heart Rate (RR), Root Mean Square of Standard Deviation of R to R intervals (RMSSD), proportion of R to R intervals larger than 50 msec (pNN50), and Triangular Index of R to R intervals.

### Statistical procedure

Changes in the HRV between the two selected therapy sessions and within each session between the beginning and the end of each recording were analyzed using 2-way repeated measure ANOVA. Measurements related to the first meeting of therapy of phase 2 are indicated by “session 1” and measurements at the end of the session of phase 2 is indicated by “session 2”. For each of these sessions, a measurement was taken at the beginning of the session (indicated by “Measurement 1”) and another at the end of the session (indicated by “Measurement 2”). There is about 25 minutes delay between “Measurement 1” and “Measurement 2” during which the patient was asked to re-experience traumatizing events – this time frame was not analyzed.

Statistical analyses combine two within-subjects factors with two levels; “Session” and “Measurement”. Additionally, other between-subjects factors and independent variables described in the previous section were included. All the analyses were carried out with General Linear Model (GLM) module of SPSS© (version 17) by using the statistics of Greenhouse-Geisser.

The existence of corrupted R to R series and/or incomplete data associated with the statistical method used (within-subjects measurement) implies that the number of subjects included in the statistical analyses was lower than 100, and varies depending on the type of analysis. R to R and demographic data are available as underlying data ^35^.

## Results

Significant changes in HR and HRV were observed. HR was higher by 3.4 beat per minute (bpm) in session 1 compared to session 2 (F(1,55)=4.99; p=0.029). Within sessions HR increased by 1.6 bpm (F(1,55)=23.53; p<0.001). There was no interaction between these two factors.

**Figure 1.**
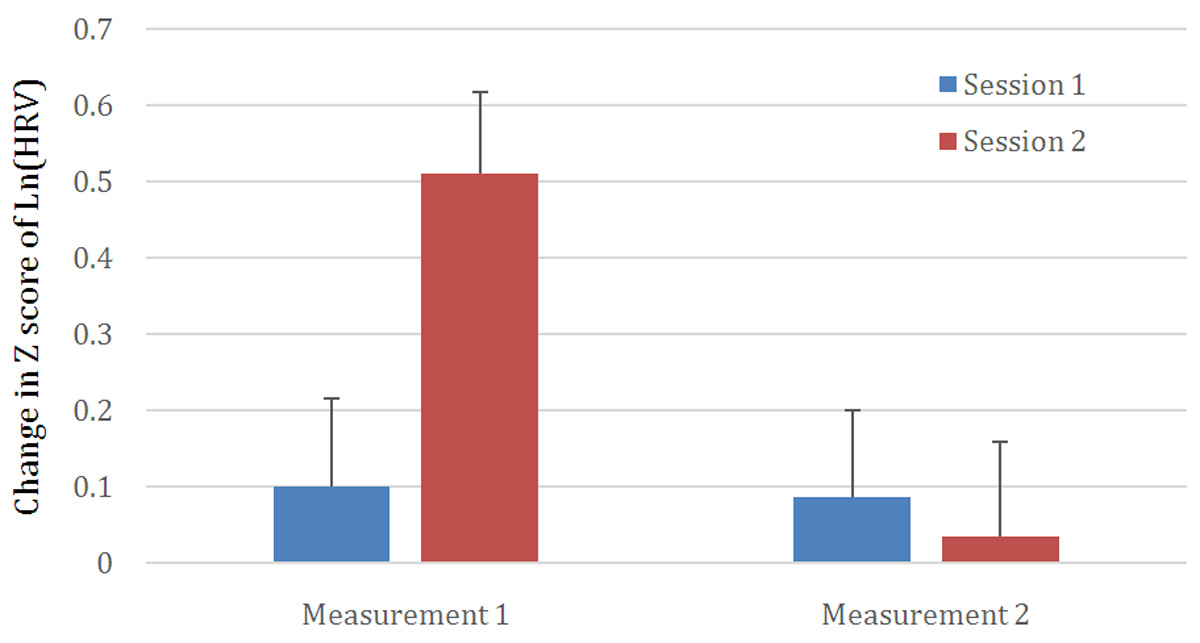
Changes in Z scores of Ln(HRV) (Ln for Napierian Logarithm) for the first and last sessions (“session”) of the therapy and within-session between the first 5 minutes (Measurement 1) and the last 5 minutes (Measurement 1) of a given session.* Depicts statistically significant differences (p < 0.05). Significant changes between measurements 1 and 2 are observed for session 2 but not for session 1. See results for more details.

Globally, total HRV estimated in the frequency domain showed significant changes as well. Within a session, HRV decrease was significant (F(1,55)=10.97; p=0.002). The total quantity of transformed HRV decreased by 0.245 points between the beginning and the end of the therapy but failed to reach significance. The interaction between the two factors was significant (D=13.32; F(1,55)=13.32; p=0.001). As shown in figure 1, this is due to the decrease in HRV between Measurement 1 and Measurement 2 being relatively large during the second session (0.476 points; p<0.05), but relatively low for session 1 (0.014 points; ns). Table 2 summarizes mean HRV values and standard errors of the mean. Figure 1 summarizes the variations in HRV based on the two factors – the Z score of Ln(HRV) was plotted where the difference was most striking.

**Table 2.** Mean heart rate variability (HRV) and standard error of the mean (in parenthesis) for all the sessions and measurements.

	Measurement 1	Measurement 2
Session 1	9834 (1329)	10907 (1887)
Session 2	7889 (939)	10602 (1124)

All other analyses of measurements of HRV obtained in the frequency domain (LF, HF, LF/HF) or time domain (Root Mean Square of Standard Deviation of R to R intervals (RMSSD), proportion of R to R intervals larger than 50 msec (pNN50), and Triangular Index of R to R intervals) did not lead to significant differences. Additional inclusion of factors (“Clinical Opinion”, “Sex” as between-subject factor, “Age”, “Number of days between Session 1 and Session 2”, “Mean number of meetings” and “Time between the two sessions” as covariates in between-subject factor) in the ANOVA did not lead to significant differences and did not modify the level of significance of the differences mentioned above. Table 3 shows the spectral LF and HF values for the different sessions and measurements

**Table 3.** Mean high-frequency (HF) and low frequency (LF) spectral values and standard error of the mean (in parenthesis) in ms ^2^/hz for all the sessions and measures.

LF	Measurement 1	Measurement 2	HF	Measurement 1	Measurement 2
Session 1	9637 (1321)	10694 (1876)	Session 1	197 (21)	214 (32)
Session 2	10972 (1102)	10409 (1117)	Session 2	231 (23)	194 (18)

## Discussion

The present study demonstrates an effect of biofeedback therapeutic interventions both in terms of heart rhythm and heart rhythm variability measurements. Subjects’ HR showed a significant decrease between session 1 and session 2 which could indicate reduced chronic stress. The reduction in the average HR in session 2 compared to session 1 can be interpreted as an effect of therapeutic interventions.

Moreover the patients’ average HR increased between the beginning and the end of each of the two sessions. This increase in HR is consistent with the model of Thayer ^18^: the patient experiences a change of emotional state due to the recall of the traumatic experience, and the induced stress leads to an increase in HR.

The analysis of the modifications of HRV partially confirms this interpretation. At the onset of session 2, patients had higher HRV than at the onset of session 1, which indicates larger parasympathetic influences towards the end of the therapy. Also, in the general population, HRV tends to be lower in patients compared to controls ^39^. In the task force of the European Society of Cardiology and the North American Society of Pacing Electrophysiology ^40^, HRV of control subjects in decubitus dorsal at rest over 5 minutes were 3466 ms ^2^/hz (± 1018 ms ^2^/hz). Measurements for the present study were approximately three times lower which could mean that HRV is close to its minimum. 1085 ms ^2^/hz (standard error of the mean (s.e.) 1329 ms ^2^/hz) were calculated for “Session 1-Measurement 1”, 1094 ms ^2^/hz (s.e. 1887 ms ^2^/hz) for “Session 1-Measurement 2”,1195 ms ^2^/hz (s.e. 939 ms ^2^/hz) for “Session 2-Measurement 1” and 1080 ms ^2^/hz (s.e. 1123 ms ^2^/hz) of “Session 2-Measurement 2”. A possible interpretation for the reduction in HRV within session 2 (and not within session 1), is that the HRV at the onset of session 2 was high enough to allow for a reduction associated with the emotional trauma recall. This was not the case in session 1 where the initial HRV was lower than in session 2 and thus might not have allowed for further reduction in HRV induced by trauma recall.

Our results have limitations. Although the collection of patients' adverse experiences revolves around the same theme, there might be some heterogeneity in the ACE reported in each session. This could complicate the comparison of sessions 1 and 2. However, we believe the disparity of intensity in adverse experiences reported in different sessions averages out across the patient population. In addition, there might have been non-responders and outlier patients. ANOVA has poor resistance to outliers, so if there were outliers, their presence was not prominent enough to invalidate our results.

Differences in HRV total power but not in the Low Frequency (LF) and High Frequency (HF) bands of the HRV were observed. The absence of an effect on HF and LF across conditions could be explained by the important inter-individual variability. HF values were weak (average of all conditions 220 ms ^2^/hz) compared to LF values (average of all conditions 1123 ms ^2^/hz). This means that the major part of the total HRV power was due to the LF component and the LF coefficient of variation was large (ranging between 1.07 and 1.94). Finally, differences of HRV between sessions and measurement times, calculated at the individual level, ranged between -1105 ms ^2^/hz and +1151 ms ^2^/hz which means that irrespective of the comparison, there were almost as many patients whose HRV varies in one direction as ones whose HRV varies in the opposite direction.

The absence of a control group and the naturalistic conditions of this retrospective study, carried out with the constraints imposed by clinical out-patient medical practice, are not ideal and resulted in large inter-individual measure variances. In this retrospective study, a large number of variables not included in the analysis might also have influenced outcome measures. For example, the decision to follow the therapy could have been accompanied by a change in lifestyle (i.e. general improvement of the hygiene of life), which may affect both HR and HRV measures. In addition, time alone could have been responsible for changes in HRV. As this study has been conceived *a posteriori*, such variables could not be controlled. However, the absence of statistical effects associated with biographical variables indicates that our results are robust across age and gender.

The intra-individual differences in the emotional reactivity following the evocation of the traumatic memory were difficult to standardize. One possible solution could be the consideration of an individual cardiovascular reactivity, which may be modeled as influenced by several independent factors ^41^. One of these factors would depend on individual physiological variables and be independent of the nature and the intensity of the emotional trauma evoked during therapy. This factor could be estimated separately using simple tests which have been used to establish relationships between the variations of HRV and the ability to regulate emotions ^6^. Other factors, such as the intensity of the trauma and the type of trauma could also influence cardiac reactivity. This multi-factor type of modeling could potentially help to reduce and understand inter-subject variability and lead to HR and HRV measures with diagnostic and therapeutic value.

In this protocol which includes two therapeutic components; HRV biofeedback and intero-nociceptive exposure, it is impossible to distinguish the impact of one component versus the other. The hypothesis was that both components are important and that it is the combination of the two which maximizes the therapeutic effect. Further studies will be necessary to investigate this hypothesis.

The analysis of HRV is a simple and non-invasive method to quantify the activity of the autonomous nervous system. The sympathetic-parasympathetic balance of patients having undergone important traumas is modified in favor of sympathetic influences. This study suggests that interoception exposure therapy – combined with biofeedback - was able to increase parasympathetic influences. Furthermore, progressive reduction in the cardiac rhythm and an increase in HRV at rest over a period of a few months were demonstrated. It is important to note that these variations were independent of the disorder diagnosed by the Psychiatrist, therefore the HRV might be considered as a general indicator of health. These results warrant further investigation of both therapeutic components (HRV biofeedback and intero-nociceptive exposure) and their comparison to other types of interventions.

## Data availability

### Underlying data

Zenodo: R-R HRV data from Biofeedback on 100 patients. http://doi.org/10.5281/zenodo.3703130 ^35^

This project contains the following underlying data:

**-**Archive_RR_All_subjects (folder containing R – R interval data for all participants as .txt files. Participants can be identified using the ID (e.g. n1799) in the file name)

**-**biographic_data.txt (Demographic data for participants)

### Extended data

Zenodo: R-R HRV data from Biofeedback on 100 patients. http://doi.org/10.5281/zenodo.3703130 ^35^

This project contains the following extended data:

- info_sheet.docx (Study data collection form, English)
- info_sheet_fr.docx (Study data collection form, French)

Data are available under the terms of the Creative Commons Attribution 4.0 International license (CC-BY 4.0).
