# Supplementary material for: Heart rate variability biofeedback intero-nociceptive emotion exposure therapy for adverse childhood experiences
Source: F1000Res. 2022 Apr 19;9:326. Originally published 2020 May 4. [Version 2] doi: 10.12688/f1000research.20776.2 (PMC9034170; doi:10.12688/f1000research.20776.2)
Supplement: Peer review history

# Peer review: Reviewer response for version 2

**DOI:** 10.5256/f1000research.123066.r135093

**Published:** 2022-04-22

**Authors:** Nele De Witte

**Affiliations:** 1 Thomas More University of Applied Sciences, Flanders, Belgium

Thank you very much for your thorough response and revision of the article. I have no further comments.

Is the work clearly and accurately presented and does it cite the current literature?

Yes

If applicable, is the statistical analysis and its interpretation appropriate?

Yes

Are all the source data underlying the results available to ensure full reproducibility?

Yes

Is the study design appropriate and is the work technically sound?

Partly

Are the conclusions drawn adequately supported by the results?

Partly

Are sufficient details of methods and analysis provided to allow replication by others?

Partly

Reviewer Expertise:

Biofeedback, emotion regulation, HRV, e-mental health, wearables. I am not an expert in the treatment of ACE and trauma so I would recommend the relevant sections (e.g.,  ACE therapy with interoceptive component) to be read by an expert in this field.

I confirm that I have read this submission and believe that I have an appropriate level of expertise to confirm that it is of an acceptable scientific standard.

# Peer review: Reviewer response for version 1

**DOI:** 10.5256/f1000research.22851.r75430

**Published:** 2020-12-16

**Authors:** Nele De Witte

**Affiliations:** 1 Thomas More University of Applied Sciences, Flanders, Belgium

The current retrospective non-controlled study aimed to investigate whether the combination of a therapeutic intervention (including emotion exposure and biofeedback) influenced HR and HRV in outpatients with ACE. HR increased during the sessions but decreased from session 1 to session 2. HRV at the beginning of the session increased from session 1 to session 2. While the fact that this is a study in naturalistic circumstances can be a strength, since we are often limited to controlled research in laboratory circumstances, this also causes severe limitations in the design and interpretation of the results. Importantly, we are lacking a control group, which severely impedes interpretation. Additionally, large individual differences occurred and we would need more information about the sample to be able to interpret these. I would also be interested in seeing additional non-physiological outcome measures of the study or information about treatment effectiveness. The manuscripts requires the addition of a lot more information and the writing style and representation of the findings can be improved. However, I do think that the study could provide interesting information to the field if these improvements are made.

**Abstract **

- “Psychiatric patients with adverse childhood experiences (ACE) tend to be dysfunctional in the interoceptive part of their emotional experience.”
  - I would suggest to state that patients or individuals have disfunctions and not that they are dysfunctional. This is a more respectful and correct representation.

**Introduction**

 General flow and readability: I would like to see some clarity in the definitions and presented research findings.The flow can also be improved. Subsections of the introduction appear to stand on their own. Some additional proofreading for the English language is also needed in the whole manuscript (e.g., “Interoception require the interplay between perception of body states and cognitive appraisal of these states to inform emotional experience and motivating regulatory behavior”).

 Specific comments:

- “Heart Rate Variability reflects an individual’s ability to adaptively cope with stress”
  - HRV is a concept that can reflect many things, including emotion regulation, but also stress, general resilience, mental illness, … (also depending on whether you are measuring resting HRV or HRV responses). So please provide some more nuance or turn the sentence around (saying that an individual’s ability to adaptively cope with stress is associated with heart Rate Variability).
- Please add a clear definition of HRV and ACE and add the abbreviation to the word when it is used for the first time. Subsequently, you should just use the abbreviation in the rest of the manuscript (please also do this for any other abbreviations you use). Also specify when you are talking about resting HRV or the HRV response in relation to certain stressors/paradigms/….  
- “Adverse childhood experiences (ACEs) are ubiquitous among the adult patient population”
  - Could you be more specific? Are their prevalence rates available?
- Please define interoception
- “In fact, most psychiatric disorders are sustained by a type of interoceptive phobia”
  - Please clarify
- Neurophysiological impairments due to ACEs have been shown to be reversible
  - Which neurophysiological impairments are you referring to here?

**Methods section**

 Please follow a more traditional sequence in the methods section, starting with the sample.

 Specific comments:

- Were participants instructed just notice bodily sensation or exert control over them?
- Consider to use “their” instead of “his/her”
- Do I understand correctly that each session of phase 2 worked with different traumatic experiences? Is it therefore possible to compare the first and last session to one another? Were you able to investigate whether a pattern emerged over different sessions or whether any change between the first and last session was possibly due to the difference in content (perhaps more difficult traumatic instances were also handled in the first session or the other way around?).
- “Patient who required psychiatric medical treatment (therapist assessment) were excluded from the study”
  - Does this refer to receiving medication or having a diagnosis other than PTSD. It isn’t entirely clear whether participants have received any formal diagnoses (if so, please specify which) or what the exact treatment context was. I was also wondering whether any underlying details on the ACE were available (how many ACEs were experienced? Severity/burden? Were there any questionnaires used?)
  - I noticed that the data collection section does have some details on diagnoses. Please add these to the sample section and please included how many individuals were suffering from each disorder. Could you also detail what the “clinical assessment” contains (is it based on a diagnostic interview, questionnaire,…).
- Could you specify what individuals were exactly doing during the HRV measurement? Were they sitting, standing, lying down? Were they resting or still actively working with the traumatic experiences?
- Is there any way to also report on the subjective effects of the intervention as research shows us that physiological and self-report data doesn’t always agree and it would be interested to have both indicators available.

Results

- Please report all results of the statistical analyses correctly (e.g., *F *(2, 54) = 4.643, *p* = .014.). You report using different HRV measurements (e.g., RMSSD, HF,…), please report which measurement you are talking about in the results. Be consistent in your terminology.
- Please add significance levels to the figure and explain a bit more what it shows.
- Where there any outliers that influenced results or were there non-responders?

Discussion

- How do you explain that your HRV values were lower than those of the task force of the European Society of Cardiology and the North American Society of Pacing Electrophysiology?
- Is there any psychophysiological data from other studies on emotional trauma recall (with or without an intervention) to compare the current data with?
- You mention very large individual differences in the effect of the intervention. Does this allow us to draw general conclusions?
- “However, the absence of statistical effects associated with biographical variables indicates that these types of effects are relatively unlikely.”
  - Please explain how you see this?
- “This study shows that interoception exposure therapy – combined with biofeedback - was able to increase parasympathetic influences”
  - This is a very strong conclusion, given that it is a non-controlled study with a lot of individual differences. Please add some nuance to this statement.
- In the concluding paragraph, the authors state “HRV at rest” does this refer to the first measurement of each sessions (or both or…?).

Is the work clearly and accurately presented and does it cite the current literature?

Yes

If applicable, is the statistical analysis and its interpretation appropriate?

Yes

Are all the source data underlying the results available to ensure full reproducibility?

Yes

Is the study design appropriate and is the work technically sound?

Partly

Are the conclusions drawn adequately supported by the results?

Partly

Are sufficient details of methods and analysis provided to allow replication by others?

Partly

Reviewer Expertise:

Biofeedback, emotion regulation, HRV, e-mental health, wearables. I am not an expert in the treatment of ACE and trauma so I would recommend the relevant sections (e.g.,  ACE therapy with interoceptive component) to be read by an expert in this field.

I confirm that I have read this submission and believe that I have an appropriate level of expertise to confirm that it is of an acceptable scientific standard, however I have significant reservations, as outlined above.

# Response

**Published:** 2022-03-31

**Authors:** Arnaud Delorme

Reviewer's suggestion: The current retrospective non-controlled study aimed to investigate whether the combination of a therapeutic intervention (including emotion exposure and biofeedback) influenced HR and HRV in outpatients with ACE. HR increased during the sessions but decreased from session 1 to session 2. HRV at the beginning of the session increased from session 1 to session 2. While the fact that this is a study in naturalistic circumstances can be a strength, since we are often limited to controlled research in laboratory circumstances, this also causes severe limitations in the design and interpretation of the results. Importantly, we are lacking a control group, which severely impedes interpretation. Additionally, large individual differences occurred and we would need more information about the sample to be able to interpret these. I would also be interested in seeing additional non-physiological outcome measures of the study or information about treatment effectiveness. The manuscripts requires the addition of a lot more information and the writing style and representation of the findings can be improved. However, I do think that the study could provide interesting information to the field if these improvements are made.

 Author's comment: We agree that the study lacks a control group. Since it is a retrospective clinical study, a control group was not possible. Still, we believe that the result of changes in patients' HRV over the course of the therapy is of scientific interest. We have clarified the method section and reorganized it. We have also worked on the writing style and remade the figure. 

 Reviewer's suggestion: "Psychiatric patients with adverse childhood experiences (ACE) tend to be dysfunctional in the interoceptive part of their emotional experience." I would suggest to state that patients or individuals have dysfunctions and not that they are dysfunctional. This is a more respectful and correct representation.

 Author's comment: The text has been changed.

 Reviewer's suggestion: "Heart Rate Variability reflects an individual's ability to adaptively cope with stress." HRV is a concept that can reflect many things, including emotion regulation, but also stress, general resilience, mental illness, … (also depending on whether you are measuring resting HRV or HRV responses). So please provide some more nuance or turn the sentence around (saying that an individual's ability to adaptively cope with stress is associated with heart Rate Variability).

 Author's comment: We have changed the text accordingly and provided more nuance to this sentence.

 Reviewer's suggestion: Please add a clear definition of HRV and ACE and add the abbreviation to the word when it is used for the first time. Subsequently, you should just use the abbreviation in the rest of the manuscript (please also do this for any other abbreviations you use). Also specify when you are talking about resting HRV or the HRV response in relation to certain stressors/paradigms/…. 

 Author's comment: We are now clearly defining HRV and ACE and differentiate between resting HRV and HRV response. In our manuscript, we are dealing with resting HRV, but by HRV response, we mean changes in HRV over the course of the therapy.

 Reviewer's suggestion: "Adverse childhood experiences (ACEs) are ubiquitous among the adult patient population". Could you be more specific? Are their prevalence rates available?

 Author's comment: We have added the prevalence rate and a citation. 

 Reviewer's suggestion: Please define interoception

 Author's comment: We are now defining interoception. 

 Reviewer's suggestion: "In fact, most psychiatric disorders are sustained by a type of interoceptive phobia." Please clarify.

 Author's comment: We have clarified this sentence. 

 Reviewer's suggestion: "Neurophysiological impairments due to ACEs have been shown to be reversible." Which neurophysiological impairments are you referring to here?

 Author's comment: We were referring to changes in hippocampal volume. We have clarified this sentence. 

 Reviewer's suggestion: Please follow a more traditional sequence in the methods section, starting with the sample.

 Author's comment: We have reorganized the method section and now start with describing our sample. 

 Reviewer's question: Were participants instructed just notice bodily sensation or exert control over them?

 Author's comment: They were instructed just to notice them. We have clarified this point. 

 Reviewer's suggestion: Consider to use "their" instead of "his/her"

 Author's comment: We have replaced all instances of "his/her" with "their"

 Reviewer's question: Do I understand correctly that each session of phase 2 worked with different traumatic experiences? Is it therefore possible to compare the first and last session to one another? Were you able to investigate whether a pattern emerged over different sessions or whether any change between the first and last session was possibly due to the difference in content (perhaps more difficult traumatic instances were also handled in the first session or the other way around?).

 Author's comment: Usually, patients' adverse experiences revolve around the same theme. The reviewer correctly mentions that there might be some heterogeneity in the ACE reported in each session. We believe this averages out across the patient population, but we have added this limitation to the discussion.

 Reviewer's question: "Patient who required psychiatric medical treatment (therapist assessment) were excluded from the study." Does this refer to receiving medication or having a diagnosis other than PTSD. It isn't entirely clear whether participants have received any formal diagnoses (if so, please specify which) or what the exact treatment context was. I was also wondering whether any underlying details on the ACE were available (how many ACEs were experienced? Severity/burden? Were there any questionnaires used?)

 Author's comment: This refers to patients receiving medication. Among others, questionnaires DES (Dissociative Experience Scale) and PCL-S (PTSD checklist – specific) were used with patients, although the diagnosis was based on the expertise of the clinician. We have clarified this section.

 Reviewer's suggestion: I noticed that the data collection section does have some details on diagnoses. Please add these to the sample section and please include how many individuals were suffering from each disorder. Could you also detail what the "clinical assessment" contains (is it based on a diagnostic interview, questionnaire,…).

 Author's comment: We have moved the section as advised. We have also clarified the clinical diagnosis. 

 Reviewer's suggestion: Could you specify what individuals were exactly doing during the HRV measurement? Were they sitting, standing, lying down? Were they resting or still actively working with the traumatic experiences?

 Author's comment: All patients were sitting at rest. We have clarified this point.

 Reviewer's suggestion: Is there any way to also report on the subjective effects of the intervention as research shows us that physiological and self-report data doesn't always agree and it would be interesting to have both indicators available.

 Author's comment: this is an interesting comment. The current data collection scheme includes the subjective assessment of both the patient and the therapist, who, after a given number of sessions, which might be different for each patient, agreed that the therapy was complete. 

 Reviewer’s suggestion: Please report all results of the statistical analyses correctly (e.g., *F *(2, 54) = 4.643, *p* = .014.). You report using different HRV measurements (e.g., RMSSD, HF,…), please report which measurement you are talking about in the results. Be consistent in your terminology.

 Author's comment: We have changed statistical reporting. We have also clarified the measures being reported. 

 Reviewer's suggestion: Please add significance levels to the figure and explain a bit more what it shows.

 Author's comment: We have added significance to the figure and additional details to its caption.

 Reviewer's question: Where there any outliers that influenced results or were there non-responders?

 Author's comment: This is a detailed analysis we did not perform. We can expect that there were non-responders and outliers, although based on the age of the data, it would take considerable effort to find the number of non-responders, and we do not think it would necessarily be informative – observing no response does not imply that the patient did not respond to the treatment. It might be possible that for a given patient with no increase in HRV, the coupling of emotional traumas with HRV is weaker. ANOVA has poor resistance to outliers, so if there were outliers, their presence was not prominent enough to invalidate our results. We have added a comment to the discussion.

 Reviewer's question: How do you explain that your HRV values were lower than those of the task force of the European Society of Cardiology and the North American Society of Pacing Electrophysiology?

 Author's comment: Low HRV is associated with stress, and the data from the European Society of Cardiology and the North American Society of Pacing Electrophysiology were for control participants. It is to be expected that HRV of patients, in general, would be lower than control participants. We have added that interpretation to the manuscript.

 Reviewer's question: Is there any psychophysiological data from other studies on emotional trauma recall (with or without an intervention) to compare the current data with?

 Author's comment: We are not aware of studies on emotional trauma recall using HRV that report the measure that we are reporting for detailed comparisons. We have added a citation to a recent study on the subject, but the data is not available. By contrast, we have made our data available. This study is consistent with decreased HRV in the population of ACE patients. 

 Reviewer's question: You mention very large individual differences in the effect of the intervention. Does this allow us to draw general conclusions?

 Author's comment: HRV is a noisy physiological measure. Large differences in HRV are common in the population, with centenarians having HRV comparable to 20-year-old individuals (despite the well-established decrease in HRV with age). Therefore, large differences in HRV response to treatment are also expected. The origin of HRV inter-individual differences is unknown to our knowledge.

 Reviewer's question: "However, the absence of statistical effects associated with biographical variables indicates that these types of effects are relatively unlikely." Please explain how you see this?

 Author's comment: We have rephrased that section to clarify this statement.

 Reviewer's suggestion: "This study shows that interoception exposure therapy – combined with biofeedback - was able to increase parasympathetic influences" This is a very strong conclusion, given that it is a non-controlled study with a lot of individual differences. Please add some nuance to this statement.

 Author's comment: We have added nuance to that statement.

 Reviewer's question: In the concluding paragraph, the authors state "HRV at rest" does this refer to the first measurement of each sessions (or both or…?)

 Author's comment: This refers to both measurements. We have clarified this in the method section.

# Peer review: Reviewer response for version 1

**DOI:** 10.5256/f1000research.22851.r72051

**Published:** 2020-11-02

**Authors:** Vedat Şar

**Affiliations:** 1 Department of Psychiatry, Koç University School of Medicine, Istanbul, Turkey

This study is based on the hypothesis that psychiatric patients with adverse childhood experiences (ACE) tend to be dysfunctional in the interoceptive dimension of the

 emotional experience which is characterized by diminished sympathovagal balance. A sufficient number of participants was subjected to exposure related to their ACE while taking the heart rate variability (HRV) to monitor the effect of the intervention. The latter covered also biofeedback sessions. A significant increase in HRV and decrease in heart rate were obtained at the end.

 The strength of this study is the hypothesis which is based on a firm theoretical basis and the transdiagnostic approach. The weakness of the study is lack of long-term follow up.

 This is a useful and well written study. To strengthen the paper, the authors should also address polyvagal theory (Porges) shortly to enrich the paper. Another subject to address is the place of HRV in the literature of PTSD that possible differences between effects of adverse childhood experiences and adult types of traumatization on HRV is dealt with.

Is the work clearly and accurately presented and does it cite the current literature?

Yes

If applicable, is the statistical analysis and its interpretation appropriate?

Yes

Are all the source data underlying the results available to ensure full reproducibility?

Yes

Is the study design appropriate and is the work technically sound?

Yes

Are the conclusions drawn adequately supported by the results?

Yes

Are sufficient details of methods and analysis provided to allow replication by others?

Yes

Reviewer Expertise:

PTSD, dissociative disorders, borderline personality disorder, functional neurological disorders, epidemiology, depression

I confirm that I have read this submission and believe that I have an appropriate level of expertise to confirm that it is of an acceptable scientific standard.
